# Using ChatGPT to navigate ambivalent and contradictory research findings on artificial intelligence

**DOI:** 10.3389/frai.2023.1195797

**Published:** 2023-07-27

**Authors:** Shahab Saquib Sohail, Dag Øivind Madsen, Yassine Himeur, Maheen Ashraf

**Affiliations:** ^1^Department of Computer Science and Engineering, School of Engineering Sciences and Technology, Jamia Hamdard, New Delhi, India; ^2^USN School of Business, University of South-Eastern Norway, Hønefoss, Norway; ^3^College of Engineering and Information Technology, University of Dubai, Dubai, United Arab Emirates

**Keywords:** ChatGPT, artificial intelligence, research findings, reaching consensus, perspective

## Abstract

With the rapid development and integration of AI in various domains, understanding the nuances of AI research has become critical for policymakers, researchers, and practitioners. However, the results are vast and diverse and even can be contradictory or ambivalent, presenting a significant challenge for individuals seeking to grasp and synthesize the findings. This perspective paper discusses the ambivalent and contradictory research findings in the literature on artificial intelligence (AI) and explores whether ChatGPT can be used to navigate and make sense of the AI literature.

## 1. Introduction

The growth and expansion of scientific research have played an essential role in developing new innovative tools and technologies that address the needs of society and individuals (Laudan, 1978; Bornmann and Mutz, 2015). Diverse research findings provide a platform and foundation for researchers to build on and utilize to advance our knowledge. However, when the results of two different scientific studies are ambivalent and contradictory, it raises the question of which results should be considered correct and how to arrive at a conclusion. Currently, there is a lack of clear guidance on how to address this issue of ambivalent and contradictory findings. This is currently the case in the literature on Artificial Intelligence (AI), an area of research that has gained increasing mainstream attention and appeal in recent months with the launch of ChatGPT and similar AI-based chatbots that can quickly generate large amounts of text on a wide variety of topics (Gordijn and Have, 2023).

However, many of the research studies that have been reported in the field of AI in recent years showcase conflicting results. These ambivalent and contradictory findings on AI and technology can lead us to believe in the existence of certain shortcomings in the research. For instance, the progress made in AI research has given rise to ethical concerns, such as the issue of determining fault in the event of an accident between an autonomous vehicle and a human-driven vehicle. In one of the studies published in Nature Human Behavior, Awad et al. (2020) claim that the driver is to be blamed. In contrast, another study blames machine-driven vehicles (Franklin et al., 2021).

Similarly, researchers contradict each other on the personalization of Google searches. For example, Pariser states in his book “The filter bubble: What the Internet is hiding from you” (Pariser, 2011) that different people receive different search results depending on the user's search history. In other words, the use of personalization algorithms creates unique information universes for each and every one of us. To illustrate how this happens in practice, Pariser had two of his friends google the keyword “BP” and found that one got links about investment opportunities in the company BP. At the same time, the other received content about the oil spill.

Contrary to this, Haim and his team (Haim et al., 2018) argue that no such events exist. In this case, they had four volunteers with entirely different preferences who searched three terms “taxation”, “Germany” and “Alstom”. Surprisingly, they observed very little difference (2.5%) in the search results, leading them to believe there was no personalization. Similarly, Krafft et al. (2019) find little room for the personalization of Google search results.

## 2. Filter bubbles

The conundrum of the existence of filter bubbles shaping search engines, online shopping, and social networking sites adds more instances of ambivalent and contradictory findings. As Sunstein explains in his book “#Republic: Divided democracy in the age of social media”, social media sorts people into groups with similar opinions, hence polarizing the online community and creating echo chambers that amplify their existing views (Sunstein, 2018). A recent study published in the Nordicom Review by Dahlgren (2021) provides a list of counterarguments to the filter bubble thesis and claims that the filter bubble thesis posits “a special kind of political human who has opinions that are strong, but at the same time highly malleable”. In contrast, Dahlgren (2021) argues that the evidence often contradicts what the actual filter bubble thesis predicts. He also points out that throughout history, there have always been people who have been skeptical of new media and technologies (e.g., the printing press), focusing mostly on the negative effects.

To some extent, a similar “moral panic” is taking place right now as commentators are discussing what impact generative AI tools such as ChatGPT will have on the education system and young minds, the scientific community, as well as businesses and work life in general (Graham, 2022; Stokel-Walker, 2022; Pavlik, 2023; Stokel-Walker and Van Noorden, 2023). The research literature on the effects of ChatGPT is still in its infancy, and while some studies have shown promising results in the chatbot's ability to answer exams on topics such as medicine (Fijačko et al., 2023; Kung et al., 2023; Mogali, 2023), law (Choi et al., 2023), and business (Terwiesch, 2023; Wood et al., 2023), it does not do as well answering questions on topics such as mathematics (Frieder et al., 2023). Generally, findings indicate that it does typically worse on answering a question related to specific areas than general and broader issues (Szabo, 2023). Many commentators remain skeptical of ChatGPT, and Borji (2023) has already curated an archive of ChatGPT failures. Therefore, already at this early point in the life of ChatGPT there is a growing body of ambivalent and contradictory research findings.

Another area of research that shows contradictory findings is related to the performance effects of AI in the business and organizational world. Many organizations sense an opportunity and seek to exploit AI to improve efficiency and reduce costs (Hassani et al., 2020). While many companies have adopted and applied AI-based tools and technologies, the failure is reportedly quite high, sometimes up to 50% (Business Wire, 2019). Although many factors are potentially at play, it is straightforward to deduce it can be hard to realize the benefits of AI, and that the results fall short of the likely inflated expectations. Sodhi et al. (2022) argue that companies that adopt emerging technologies with unrealistic expectations “may find themselves at the bleeding rather than at the leading edge of technology” (p. 2,534). Additionally, in some situations, it is possible to get unclear or contradictory outcomes, e.g., when normative assumptions used by AI algorithms are clarified (Cooper, 2020). Again, this is another proof that AI has contradictory forces, and fairly interpreting the results of AI-based systems is still challenging.

Considering the above scenarios, the question that arises is: What criteria can be used to draw an affirmative conclusion? Should the reputation of journals or the authors be considered a benchmark for research? To that end, we urge the scientific community to develop new evaluation protocols and processes that can adequately interpret and make sense of the conflicting results of AI-based studies and correctly identify the reasons behind the contradictions achieved in similar studies, for example, if there is a study that provides a different conclusion from the research published earlier. In that case, the most recent publication should cite the previous one and explain how their newfound conclusions differ from the pre-existing results and how and why they should be considered more accurate. In addition, a scientific body or a team could cross-check the issue to conclude the debate, as arriving at a consensus for these ambivalent and contradictory findings could help researchers in the future advancement of the field. Furthermore, contemplative observations made by a rigorous review system by experts with deep knowledge in the domain can serve as a possible solution to this issue.

## 3. Roles of ChatGPT in navigating ambivalent and contradictory research findings

A more radical and speculative solution would be to leverage ChatGPT to reach a consensus when faced with the ambivalent and contradictory results in the research reported on AI. There are several ways in which AI could potentially be useful in this regard.

- Synthesizing and summarizing research findings: ChatGPT could be trained on a corpus of AI research papers and used to generate summaries of key findings from multiple studies. Several studies suggest that ChatGPT is reasonably useful for carrying out tasks such as literature searches and reviews (Biswas, 2023; Dowling and Lucey, 2023; Lund et al., 2023). This could help researchers identify areas of agreement and disagreement across different studies and help highlight areas in need of more research. However, it is essential to note that some commentators have pointed out instances where ChatGPT has fabricated convincing responses containing errors and misrepresentations (Farhat et al., 2023; van Dis et al., 2023).
- Providing context and background information: ChatGPT could provide context and background information about specific AI research topics, helping researchers better understand the nuances of different studies and their findings. Again, researchers must remain vigilant and cognizant of errors and misrepresentations (van Dis et al., 2023).
- Generating hypotheses and identifying research gaps: ChatGPT could be used to generate hypotheses based on existing research findings and to identify gaps or areas in the literature where more research is needed. Recent research in the field of finance has shown that ChatGPT is useful for generating plausible research ideas (Dowling and Lucey, 2023). Hence, ChatGPT could help researchers focus their efforts and resources more effectively.
- Supporting collaboration and discussion: ChatGPT could facilitate collaboration and discussion among researchers working on similar topics or issues. As pointed out by Lin (2023), it is “a great channel to bounce ideas off”. By providing a common language and context, ChatGPT could help to reduce “silo thinking” (common in business organizations and well as universities) and promote more productive exchanges of ideas and findings. As an illustrative example of how ChatGPT could be used, one of the authors of the current paper asked ChatGPT about the existence of filter bubbles, which is one of the concepts that we discussed earlier. ChatGPT provided the following answer:

*The concept of a filter bubble refers to the idea that when we use personalized search engines, social media platforms, or other recommendation systems, we may be exposed only to information and perspectives that align with our existing beliefs and interests. This can create a situation where our worldviews become increasingly narrow, and we may miss out on important information or alternative viewpoints*.

*There is evidence to suggest that filter bubbles do exist to some extent. Studies have found that personalized search results can reinforce existing biases and that social media algorithms can lead to the propagation of false or misleading information. However, the extent to which filter bubbles affect individuals and society is still a matter of debate. Some experts argue that filter bubbles are overblown and that people are still exposed to a wide range of information from a variety of sources. Others suggest that the effects of filter bubbles may be more pronounced for certain groups, such as people with extreme political views or those who rely heavily on social media for news and information. Overall, while there is evidence to suggest that filter bubbles exist, the extent and impact of these bubbles are still a subject of ongoing research and discussion*.

However, when one of the authors prompted ChatGPT a few days later, a slightly different response was provided:

*Yes, filter bubbles exist. A filter bubble is a phenomenon where an algorithm selectively guesses what information a user would like to see based on the user's past behavior, likes, and search history. This results in the user being exposed to content that reinforces their beliefs, preferences, and biases while sheltered from opposing viewpoints and information*.

*Filter bubbles can lead to a narrow and skewed world understanding, limiting exposure to diverse opinions and ideas. This can make it harder for individuals to engage in constructive dialogue and compromise with those who hold different beliefs, leading to social and political polarization. Additionally, filter bubbles can contribute to the spread of misinformation and fake news, which can have negative consequences for society*.

*While the existence of filter bubbles is widely accepted, there is an ongoing debate about their extent and impact on individuals and society and what can be done to mitigate their effects*.

The latter response generally appears to be more supportive of the filter bubble thesis by Pariser (2011). Hence, the responses generated by ChatGPT on this topic vary, but it is unclear what factors are causing these variations. Is it because ChatGPT is continuously learning, or is it due to personalization algorithms, as was discussed earlier? For example, the responses were generated by authors living and working in different regions of the world and presumably with different search histories. It is not clear whether this shapes the responses that ChatGPT provides.

## 4. Conclusion

This perspective paper has explored ChatGPT's ability to navigate and reach a consensus on AI research findings. These results vary greatly and could be seen as inconsistent or equivocal, making it quite challenging for individuals who are trying to understand and integrate these findings. While there is reason to believe that ChatGPT and similar chatbots could help researchers navigate complex research fields such as AI, it is still too early to draw conclusions regarding this matter. Much of this is due to the rapid progression of ChatGPT and similar chatbots, as their functionalities continue to advance. Consequently, our brief paper poses more questions than it answers. Our hope is that the issues and perspectives discussed in this paper will encourage further discourse within the scientific community.

## Data availability statement

The original contributions presented in the study are included in the article/supplementary material, further inquiries can be directed to the corresponding author.

## Author contributions

All authors listed have made a substantial, direct, and intellectual contribution to the work and approved it for publication.
